# Ear-Pain, and Its Treatment

**Published:** 1887-11

**Authors:** E. Meirhof

**Affiliations:** Attendant Out-Door Department Mt. Sinai Hospital, New York


### EAR-PAIN AND ITS TREATMENT.

*By E. Meirhof, M. D., Attendant Out-Door Department Mt. Sinai Hospital, New York.*

For Daniel's Texas Medical Journal.

THE various causes giving rise to ear-pain are, I regret to state, not only overlooked, but often knowingly ignored by the general practitioner. It is true that the ignorance prevailing regarding diseases of the ear is, in a great measure, pardonable, owing to the limited clinical experience heretofore obtained during a regular course of medical education. Fortunately it is, that in the large majority, cases of ear-pain, except where bony structure is involved, are spontaneously relieved by nature, after a few hours or days. There are a number of causes in the production of ear-pain. Youth and childhood are more susceptible to pain in and about the ears than those of maturer years. Ear-pain is generally of one or two forms—reflex or inflammatory. The reflex form is oftener met with during childhood, being frequently due to the process of dentition. Occasionally the eruption of the wisdom teeth gives rise to ear-pain. When ear-pain is reflex, it is characterized by the intermittence of the pain. By day it is often absent, but at night it asserts itself sufficiently to make amends for the lost time which the patient has not spent in suffering during the day. Its non-inflammatory character may be recognized by the fact that pressure upon and around the orifice of the external auditory canal, or pulling on the auricle does not cause or increase pain. In young children, an examination of the tympanic membrane is frequently unsatisfactory, and therefore negative in its result. A sufferer from ear-pain having no fever, no discharge from the ear, the tympanic membrane translucent, with no blood vessels coursing over its surface,* no increased pain on pressure over the ear, the painful paroxysms frequently nocturnal, may generally be considered as having a reflex form of ear-pain.

The inflammatory forms of ear-pain are various. In one class of cases the pain is caused by an inflammation of the external canal, either by a furuncle, or a more diffuse inflammation of lining of the canal; sometimes impacted cerumen may give rise to pain, and of these, the furuncular form of inflammation, the practitioner is apt to meet frequently. Of the other forms of ear inflammation which give rise to pain, there are the acute catarrhal, and the acute suppurative inflammation of the middle ear; also chronic suppurative disease of the middle ear, complicated with polypi, and its extension to the mastoid cells.

As I am dealing with pain and its relief, I therefore desire to treat of pain in an abstract way, merely for the purpose of making some suggestions as to the relief of the most urgent symptom in connection with diseases of the ear.

I have found from experience, that in the majority of cases, relief can be afforded by local measures alone; although occasionally, it is necessary to resort to internal treatment. For the relief of reflex ear-pain, atropine stands first cn the list of remedies; and frequently this is all that is nccessary to subdue it. A one per cent solution is a strength commonly used. After cleansing the external auditory canal, a piece of absorbent cotton is packed into the canal. and saturated with the above solution. Cocaine seldom gives as much relief as atropine. I wish to warn the reader that atropinization has occurred, at which there need be no alarm. Another local remedy which will be found efficient, is painting the surface over the mastoid process, around auricle beneath the lobe and in front of the orifice of the canal, with a liquid, made by rubbing equal parts of camphor and chloral hydrate together; this may be repeated every two hours. The internal exhibition of such drugs as the bromides, hyosciamus, opium, etc., which lessen reflex

---

*The vascular supply of the normal drum-head is not visible to the naked eye.

excitability, may be found occasionally necessary; but the writer seldom resorts to them for the relief of reflex ear-pain.

Pain from a furuncle is best relieved by an early incision through the base of the swelling. Do not nick the boil, because this often makes matters worse; but make the incision from the base upwards, through the apex. It is the tension caused by the unyielding nature of the tissues, which makes a boil in this region, so exquisitely painful, and therefore the need of an early incision; the soreness left may be alleviated by the use of atropine. The diffuse form of inflammation of external auditory canal is often caused by a number of boils, or a diffuse cellulitis. In these cases the lumen of the canal is quite occluded, and a stream of water, hot as the patient can bear, directed to the ear about the orifice, for about ten minutes every hour or two, will materially lessen the swelling, or an incision may be made in the part most swollen, and hot water used subsequently. In directing the hot water, it is not necessary to pull on the pinna, in order to straighten the canal, as is usually done for the removal of impacted cerumen, foreign bodies, etc., but simply direct the stream against the auricle around the orifice, without touching it. If there be any fear of the hot water finding its way in the canal, a piece of absorbent cotton may be placed in the orifice if possible. It may also be necessary to administer morphine to aid in the relief of pain.

Acute catarrhal and acute suppurative inflammation of the middle ear, the general practitioner frequently meets in scarlet fever, diphtheria, measles, etc. The relief from ear pain in these cases is frequently ignored. Here, again, a solution of atropine will very often give much relief, and thereby save patients a great deal of suffering and exhaustion. An incision or puncture into the drumhead does not always relieve pain, neither is harm done if pus is not found; but much good may be done in anticipating a destructive process which may take place for pent up pus to find an exit. Inflation of the middle ear should not be neglected in connection with other treatment, as it is a necessary adjunct, whether ear disease is accompanied by pain or not. Pain, as met in chronic suppurative inflammation of the middle ear, urgently calls for relief; as this is usual evidence of the extension of the long standing inflammation to deeper portions than the soft parts; and as inflammation of the perimastoideum, and mastoid cells is generally a slower process than the acute affections already mentioned, something can be done to relieve the pain by therapeutic measures; but surgical interference is often required. The external auditory canal should be cleansed, either by cotton mops, or by a warm stream of any reliable antiseptic solution; if the patient is not very much reduced, several leeches might be applied in front of the ear; not on the mastoid process, as is often done. The constant application of a small ice bag over the mastoid region, together with the internal use of quinine and morphine will aid materially in relieving pain, etc.; but surgical interference should not be put off too long if pain, etc., is not relieved in a reasonable time.

